# Dual Organism Transcriptomics of Airway Epithelial Cells Interacting with Conidia of *Aspergillus fumigatus*

**DOI:** 10.1371/journal.pone.0020527

**Published:** 2011-05-31

**Authors:** Jean L. Oosthuizen, Pol Gomez, Jian Ruan, Tillie L. Hackett, Margo M. Moore, Darryl A. Knight, Scott J. Tebbutt

**Affiliations:** 1 UBC James Hogg Research Centre, Institute for HEART+LUNG Health, Providence Health Care, Vancouver, British Columbia, Canada; 2 Department of Anesthesiology, Pharmacology and Therapeutics, University of British Columbia, Vancouver, British Columbia, Canada; 3 Department of Biological Sciences, Simon Fraser University, Burnaby, British Columbia, Canada; 4 Division of Respiratory Medicine, Department of Medicine, University of British Columbia, Vancouver, British Columbia, Canada; University of Pittsburgh, United States of America

## Abstract

**Background:**

Given the complex nature of the responses that can occur in host-pathogen interactions, dual transcriptomics offers a powerful method of elucidating these interactions during infection. The gene expression patterns of *Aspergillus fumigatus* conidia or host cells have been reported in a number of previous studies, but each focused on only one of the interacting organisms. In the present study, we profiled simultaneously the transcriptional response of both *A. fumigatus* and human airway epithelial cells (AECs).

**Methodology:**

16HBE14o- transformed bronchial epithelial cells were incubated with *A. fumigatus* conidia at 37°C for 6 hours, followed by genome-wide transcriptome analysis using human and fungal microarrays. Differentially expressed gene lists were generated from the microarrays, from which biologically relevant themes were identified. Human and fungal candidate genes were selected for validation, using RT-qPCR, in both 16HBE14o- cells and primary AECs co-cultured with conidia.

**Principal Findings:**

We report that ontologies related to the innate immune response are activated by co-incubation with *A. fumigatus* condia, and interleukin-6 (IL-6) was confirmed to be up-regulated in primary AECs via RT-qPCR. Concomitantly, *A. fumigatus* was found to up-regulate fungal pathways involved in iron acquisition, vacuolar acidification, and formate dehydrogenase activity.

**Conclusion:**

To our knowledge, this is the first study to apply a dual organism transcriptomics approach to interactions of *A. fumigatus* conidia and human airway epithelial cells. The up-regulation of IL-6 by epithelia and simultaneous activation of several pathways by fungal conidia warrants further investigation as we seek to better understand this interaction in both health and disease. The cellular response of the airway epithelium to *A. fumigatus* is important to understand if we are to improve host-pathogen outcomes.

## Introduction

Microarray technologies have enabled unbiased gene expression profiling in host-pathogen interactions [1]. Using such technology, one can interrogate both organisms at the transcriptome level, allowing characterization of the dynamic interaction between microbe and the host environment. This includes defining mechanisms of microbe survival and the host's identification of the microbe and its subsequent clearance. Such an approach has been successfully applied to the soybean *Glycine max* and its two major parasites, *Phytophthora sojae* and *Heterodera glycines* [2], [3]. With respect to mammals, dual organism profiling was utilized by Motley *et al.* to characterize *E. coli* infection in a murine granulomatous pouch model [4]. Given the complex nature of the responses that can occur in host-pathogen interactions, dual transcriptomics offers a powerful method of elucidating the nature of interactions during infection. It also has the potential to identify differences between healthy and diseased tissues that may facilitate opportunistic infections.

*Aspergillus fumigatus* is a ubiquitous saprophytic mold [5] that is capable of causing a spectrum of diseases, particularly in patients with underlying respiratory conditions or immunodeficiency. For example, allergic bronchopulmonary aspergillosis (ABPA) is a hypersensitivity disorder caused by *A. fumigatus*, affecting up to 5% of asthmatics and 10% of cystic fibrosis patients [6], [7]. Another disease is aspergilloma, a non-invasive fungal growth in a pre-existing lung cavity, as may result from tuberculosis, sarcoidosis, or other cavitary lung diseases [8], and can occur in 10–15% of patients with such cavities [9], [10]. Invasive pulmonary aspergillosis is a life-threatening condition that may occur in immunocompromised patients, particularly those with neutropenia [11]. Fungal conidiospores (conidia) enter the airways where they germinate and undergo hyphal extension into the lung parenchyma, often followed by systemic spread.

*A. fumigatus* is dispersed as 2–3 µm haploid conidia which are estimated to be found at concentrations of 1 to 100 colony-forming units per cubic metre of air, meaning the average human inhales up to several hundred conidia each day [7]. The bronchial epithelium serves as a point of first contact and structural barrier to *A. fumigatus* infection, and hence represents an important site of interaction [9]. While earlier works demonstrated the uptake by and germination of conidia within airway cells [12], [13]–[16], the characterization of transcriptional responses to interaction is only recent. Our previous work identified the transcriptional response of the SV40 transformed human bronchial epithelial cell line, 16HBE14o-, following internalization of *A. fumigatus* conidia [17]. We showed that 16HBE14o- cells internalized 30–50% of bound conidia, and had increased levels of transcripts from genes associated with repair and inflammatory processes as a result (*e.g.*, matrix metalloproteinases, chemokines, and glutathione S-transferase) [17]. In the present study, we have investigated the interaction of conidia and the early transcriptomic response by both *A. fumigatus* conidia and primary airway epithelial cells (AECs). We hypothesize that the early cellular response of the airway epithelium to *A. fumigatus* conidia (and vice versa) is important to understand if we are to improve host-pathogen interaction outcomes.

## Methods

### Ethics approval

Ethics approval (#H0-50110) was obtained from the University of British Columbia Institutional Ethics Review Board.

### Aspergillus fumigatus strain

All experiments were performed using a green fluorescent protein (GFP) expressing strain of *A. fumigatus* derived from ATCC 13073, developed by Wasylnka and Moore [12]. Conidia were stored and prepared as previously described [17].

### 16HBE14o- cell line

16HBE14o- transformed bronchial epithelial cells were obtained from Dr. D. Gruenert (University of Vermont, Burlington, VT, USA). Important characteristics of differentiated human bronchial epithelium are retained in this cell line, including directional ion transport, formation of a monolayer, and tight junctions [18]. Cultures were maintained as previously described by Gomez *et al.* [17].

### Primary human airway epithelial cells (AECs)

Primary AECs were isolated from human lungs from donors with no pulmonary disease or smoking history but deemed unsuitable for transplantation and donated for medical research with written family consent through the International Institute for the Advancement of Medicine (Edison, NJ, USA). The ethic committees of the involved institutions approved this study. AECs were isolated as previously described [19]. Briefly, following surgical removal, lungs were washed in Custodial HTK solution (Odyssey Pharmaceutical Inc., East Hanover, NJ, USA) and placed on ice. Trachea and bronchi were then dissected into short segments and rinsed in cold PBS for blood and mucous plug removal. Epithelium was dissociated with a Pronase (1.4 mg/ml) and DNase (0.1 mg/ml) (Roche Diagnostics, USA) treatment in 100 ml minimal essential medium (MEM) for 16 hours at 4°C. Dissociated clumps were strained through 70 µm nylon mesh (Becton, Dickinson and Company, USA), incubated in MEM with 10% v/v FBS to neutralize the Pronase, and washed with MEM at 4°C. Adherent cells were grown in bronchial epithelial growth medium (BEGM; Cambrex, Walkersville, MD, USA).

### Exposure of epithelial cells to *A. fumigatus* conidia

16HBE14o- and AECs were seeded in 4-chamber Culture Slides (BD Biosciences, Franklin Lakes, NJ, USA) in 1 ml DMEM or BEGM, respectively. Cells were then grown for 3 days at 37°C with 5% CO_2_ to achieve confluency. Chamber media was replaced with 500 µl of fresh DMEM or BEGM and incubated with 10^5^ *A. fumigatus* conidia, for an average final multiplicity of infection of one conidium per cell. The cells and conidia were co-incubated at 37°C for 6 hours, and then washed three times in PBS-T to remove any conidia not bound to cells. Human cells and fungal conidia that were not exposed to each other (controls) were incubated under the same culture medium conditions as cells and conidia that were co-exposed. The 6 hour co-incubation permits comparability to previous studies that have used this time-point, and ensures that the response observed corresponds to interaction with conidia [12], [15], [17]. Beyond 6 hours, conidia have been found to germinate in culture [12].

### Analysis by confocal microscopy

Cultures were treated as above and imaged as previously described [17]. Images from all specimens were obtained using the Multiphoton Confocal Microscope System at the Core 3 Dynamic Cellular Imaging and Biophysics facility of the James Hogg Research Centre. Images were acquired using a Leica AOBS SP2 laser scanning confocal microscope (Leica, Heidelberg, Germany) with Zeiss LSM 510 software, version 3.2 (Carl Zeiss Canada Inc., Toronto, ON, Canada). A series of images were acquired in the Z-plane, allowing for three dimensional reconstruction and visualization of the cell monolayer and associated *A. fumigatus* conidia.

### RNA extraction

RNA extraction was performed using an RNeasy Mini Kit with QIAshredder (Qiagen, USA). A modification to the manufacturer's protocol for “Purification of Total RNA from Plant Cells and Tissues and Filamentous Fungi” was used. Briefly, samples were spun down, immersed in liquid nitrogen for 5 minutes, and then ground with plastic mini-pestles (DiaMed, Mississauga, ON, Canada). Tubes were heated for 2 minutes at 56°C and passed through the QIAshredder column. The RNA yield from each sample was determined using a NanoDrop™ ND-1000 spectrophotometer (Thermo Scientific, Wilmington, DE, USA). RNA integrity of the samples was determined using a 2100 Bioanalyzer (Agilent Technologies, Stockport, Cheshire, UK). The 2100 Bioanalyzer generates an RNA integrity number (RIN) that has been shown to reliably predict suitability of RNA samples for gene expression analysis [20].

### Transcriptome profiling

Microarray services were performed by the Prostate Centre Microarray Facility (Vancouver, Canada), an Agilent Certified Service Provider. Human gene expression was analyzed using Agilent Whole Human Genome Oligo Microarrays in the 4×44 K format (product number G4112F, design ID 014850, Agilent Technologies). Fungal transcriptome analysis was performed on conidia-containing samples using JCVIGR *Aspergillus fumigatus* Version 3 microarray slides (Pathogen Functional Genomics Resource Center, Rockville, MD, USA). Eight arrays representing 4 biological replicates each of cells alone and cells plus conidia were analyzed for each species. Human probes with a p-value<0.05 and fold change (FC)>1.5 were considered differentially expressed. ANOVA was used to identify fungal probes showing differential expression between the two conditions at a significance of p<0.05 and FC>1.5. All microarray data are MIAME compliant. The raw data have been deposited in a MIAME compliant database - the Gene Expression Omnibus (GEO) - and are accessible under Series GSE16628, GSE16630 and GSE16637.

### Reverse transcription quantitative PCR (RT-qPCR)

AEC RT-qPCR was performed using 20× Applied Biosystems probe-primer mix (Table S1) and TaqMan® Universal PCR Master Mix (Roche Molecular Systems, CA, USA). *A. fumigatus* RT-qPCR was performed using the 20× Integrated DNA Technologies (Coralville, IA, USA) probe-primer mix (Table S2) with Stratagene Brilliant II QPCR high ROX master mix. Both reactions were performed using an ABI Prism® 7900 Sequence Detection System. The gene glyceraldehyde 3-phosphate dehydrogenase (*gpdA*) was used as an endogenous control for *A. fumigatus* to generate a normalization factor. The relative standard curve method was applied to determine relative fold-change [21]. The genes peptidyprolyl isomerase A (PPIA) and phosphoglycerate kinase 1 (PGK1) were used for AECs, having been previously shown to be the most stably expressed genes in bronchial epithelial cells [22]. These two genes were used to generate a normalization factor by geometric averaging, and the relative standard curve method applied to determine relative fold-change [21], [23].

## Results

### Confocal microscopy analysis of primary AECs and *A. fumigatus* conidia in co-culture

**Figure 1:**
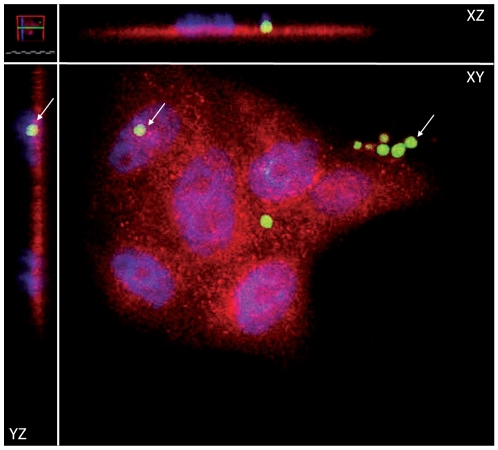
Localization of *A. fumigatus* conidia within the airway epithelial cell monolayer. GFP-expressing *A. fumigatus* conidia and primary AECs were co-incubated for 6 hours and treated with DAPI and monoclonal E-cadherin Alexa 594 antibody before visualization by confocal microscopy. Labeling of nuclei (blue) and the membrane tight junctional protein E-cadherin (red) allowed visualization of AECs. Some GFP-expressing *A. fumigatus* conidia (green) are found outside the cells, while others localize within the cell monolayer, in close association with AEC nuclei.

To assess whether co-incubation would lead to the uptake of *A. fumigatus* conidia by primary human AECs, co-cultures were first visualized using incremental focal planes, to produce a Z-stack to form a three dimensional image by confocal microscopy. As shown in Figure 1, conidia expressing GFP co-incubated with AECs could be seen adjacent both to the cell plasma membrane identified by E-cadherin staining (red staining), and also to the AEC nucleus identified by DAPI (blue staining).

### Differential gene expression in AECs following exposure to *A. fumigatus* conidia

We have previously demonstrated differential gene expression in 16HBE14o- cells following direct interaction with GFP-*A. fumigatus* conidia identified by fluorescence activated cell sorting (FACS), compared to 16HBE14o- cells within the same culture not positive for GFP- *A. fumigatus* conidia [17]. While these experiments identified differentially expressed genes from 16HBE14o- cells in direct interaction with *A. fumigatus* conidia, we could not determine the effect on the entire culture which would be representative of interaction with the airway epithelium *in vivo*.

In this study, microarray data was obtained from 16HBE14o- cells following incubation with or without *A. fumigatus* conidia. Eight human arrays, representing 4 biological replicates, were derived from cells co-incubated with and without *A. fumigatus* conidia. From this analysis we identified 255 human genes that were differentially expressed, with p<0.05 and fold change 1.5 or greater; however, no genes survived at a false discovery rate (FDR) of 0.05 (Table S3). We compared the data sets of both the current and previous studies to identify commonalities, but found only 17 genes that were common to 16HBE14o- cells that were either incubated with or directly associated with *A. fumigatus* conidia [17].

Using Ingenuity Pathway Analysis (IPA) software, 174 of 255 genes met the criteria for further analysis. Among the top networks identified by core analysis were those implicated in gene expression, infection mechanism, and cellular movement. Gene Ontology Enrichment Analysis (GOEAST) and Gene Set Enrichment Analysis (GSEA) were also conducted to complement and add support to the data obtained using IPA (Table 1 and data not shown). Again looking for commonality with previous work, we found the annotated functions and pathways of differentially expressed genes indicate that the innate immune response is one of the prevalent themes in both of the expression data sets [17].

**Table 1:** Over-represented human gene ontology (GO) terms determined by GOEAST analysis.

List of Terms Up-Regulated			
Ontology	GO ID	Term	P-value
positive regulation of glycogen biosynthetic process	GO:0045725	BP	2.54E-04
epithelial structure maintenance	GO:0010669	BP	1.08E-03
zinc ion homeostasis	GO:0055069	BP	2.04E-03
virion transport	GO:0046794	BP	7.13E-03
toxin metabolic process	GO:0009404	BP	1.24E-02
C-X-C chemokine receptor activity	GO:0016494	MF	2.14E-02

List of Terms Down-Regulated			
Ontology	GO ID	Term	P-value
transition metal ion binding	GO:0046914	MF	9.48E-10
regulation of transcription, DNA-dependent	GO:0006355	BP	1.76E-07
regulation of metabolic process	GO:0019222	BP	1.58E-06
gene expression	GO:0010467	BP	8.02E-06
cellular nitrogen compound metabolic process	GO:0034641	BP	8.31E-06
nucleus	GO:0005634	CC	5.51E-05

Differentially expressed genes from microarray analysis of 16HBE14o- cells incubated with conidia of *Aspergillus fumigatus*. Input lists consisted of 109 up-regulated and 146 down-regulated genes. (Terms: BP – biological process; MF – molecular function; CC – cellular compartment.).

To validate the changes in expression levels demonstrated by the microarray experiments, we selected eight genes to analyze by RT-qPCR. These genes were implicated by either pathway analysis or directly from the array data. Samples representing FAC sorted 16HBE14o- or unsorted AECs were used in this assay, thereby revealing whether expression differences were consistent between both cell types. The genes chemokine (C-C motif) ligand 3 (CCL3), chemokine (C-C motif) ligand 5 (CCL5), IL-6, colony stimulating factor 2 (CSF2), and matrix metallopeptidase 1 (MMP1) were chosen based on their inclusion in the gene set level data, as well as a relative strength at the single gene level. Viewed in IPA, these genes formed distinct clusters with clear relationships to the differentially expressed genes (Figure S1). These nodes are also prominent components of the annotated classifications identified in GOEAST and GSEA.

**Figure 2:**
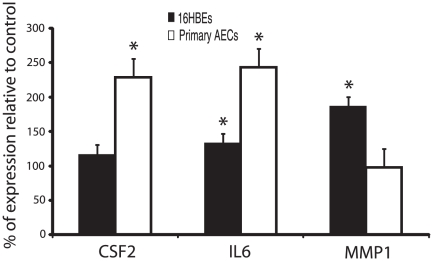
Relative mRNA expression levels of human genes obtained by RT-qPCR. RNA was obtained from four co-incubations each of 16HBE14o- (grey bars) or primary normal bronchial epithelial cells (AECs) (white bars) with conidia of *A. fumigatus*. Height of each bar represents expression of gene in co-incubated condition relative to cells alone control (mean ± SE). (* p<0.05).

Three additional targets were chosen based on their strength as among the most strongly differentially expressed, in a consistent fashion, in both experiments. These genes are zinc finger 433 (ZNF433), leucine rich repeat containing 14 (LRRC14), and DOT1-like, histone H3 methyltransferase (DOT1L). Despite relatively little information regarding function, they may nonetheless represent genes involved in the cellular response to *A. fumigatus*. The results of RT-qPCR can be seen in Figure 2. IL-6 was the only gene tested with RT-qPCR that was differentially expressed in both cell types following co-incubation with *A. fumigatus* conidia. Both MMP1 and CSF2 each achieved significant differential expression in only one of the cell types tested. CCL3, CCL5, ZNF433, DOT1L and LRRC14 failed to achieve significant differential expression in either cell type following incubation with *A. fumigatus* condia.

### Genes showing differential expression in *A. fumigatus* conidia following exposure to human bronchial epithelial cells

To gain insight into the response of *A. fumigatus* to the host cell environment, we then focused on identifying the transcriptional response of conidia to interaction with human AECs. At a significance of p<0.05 and fold change greater than 1.5, we found 150 genes that were up-regulated in conidia exposed to human bronchial epithelial cells (16HBE14o-), whereas 33 were down-regulated (Table S4). To assess any biologically relevant themes within the up-regulated gene set, GOEAST was conducted using annotated terms of the close fungal relative, *Aspergillus nidulans*.

**Figure 3:**
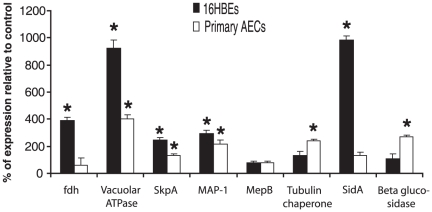
Relative mRNA expression levels of *A. fumigatus* genes as determined by RT-qPCR. RNA was obtained from four co-incubations each of *A. fumigatus* conidia with 16HBE14o- (grey bars) or primary human bronchial epithelial cells (AECs) (white bars). Height of each bar represents expression of gene in co-incubated condition relative to conida alone control (mean ± SE). (* p<0.05).

Among the most prominent annotated functions identified involved genes classified in vacuolar acidification and metallopeptidase activities. To validate the expression levels generated by the microarray, eight genes in total were chosen for further analysis with RT-qPCR (Table 2). Four of these genes, metallopeptidase *MepB*, matrix AAA protease *MAP-1*, sulphur metabolism regulator *SkpA*, and the vacuolar ATPase 98 kDa subunit, were chosen as gene targets of interest based on the significance of their GOEAST classifications. *MepB* and *Map-1* are part of the metallopeptidase classification, while *SkpA* and the Vacuolar ATPase 98 kDa subunit are associated with the vacuolar acidification gene set. The selection of the other four targets, tubulin-specific chaperone C, NAD-dependent formate dehydrogenase (*fdh*), β-glucosidase, and L-ornithine *N^5^*-oxygenase (*SidA*), was based on significance at the single gene level. We have an interest in iron metabolism by *A. fumigatus*, and siderophore-mediated iron acquisition is critical for virulence [24], [25]. These genes were tested in two different incubation types, one involving 16HBE14o- cells and the other a co-incubation with AECs (Figure 3). Interestingly, all genes achieved significance following incubation with at least one cell type, with the exception of *MepB*. The vacuolar ATPase 98 kDA subunit, *SkpA*, and *MAP-1* were significantly differentially expressed following incubations with both 16HBE14o- and AECs. Significance was achieved only in 16HBE14o- cells for *fdh* and *SidA*, whereas the tubulin-specific chaperone C and β-glucosidase were significant only in the AECs.

**Table 2:** Microarray expression data for *Aspergillus fumigatus* genes chosen for RT-qPCR.

Locus ID	Gene name *(designation)*	P-value	Fold-change
Afu6g04920	NAD-dependent formate dehydrogenase (*fdh*)	9.09E-02	1.55
Afu4g11300	vacuolar ATPase 98 kDa subunit	1.39E-02	2.51
Afu5g06060	sulfur metabolism regulator (*SkpA*)	5.44E-02	1.53
Afu2g02680	matrix AAA protease (*MAP-1*)	3.42E-02	2.53
Afu7g05930	metallopeptidase (*MepB*)	1.05E-03	2.42
Afu3g08900	tubulin-specific chaperone c, putative	4.41E-04	2.67
Afu2g07680	L-ornithine *N^5^*-oxygenase (*SidA)*	1.70E-02	2.38
Afu1g14710	Beta-glucosidase	8.35E-04	2.66

## Discussion

The gene expression patterns of both *A. fumigatus* conidia and host cells have been reported in a number of previous studies, but each with a focus on only one of the interacting organisms [17], [26], [27]. However, varying experimental conditions and cell types can make it difficult to compose a representative model of interaction that encompasses the responses of both organisms. In the present study, we profiled the transcriptional response of both *A. fumigatus* and human AECs simultaneously to better elucidate the dynamic responses between the two interacting organisms. To our knowledge, this is the first study to apply a dual organism transcriptomics approach to interactions of *A. fumigatus* conidia and human airway epithelial cells.

Previous studies have focused on the role of immune cells, primarily macrophages and neutrophils, in response to *A. fumigatus* exposure [26]–[29]. Whilst professional phagocytic cells are important in mediating fungal clearance, the airway epithelium is the first point of contact for fungal conidia within the lung and represents an important surface of interaction [30]. Indeed many *A. fumigatus*-mediated diseases such as ABPA, severe asthma with fungal sensitization (SAFS), aspergilloma, and invasive pulmonary aspergillosis are associated with the respiratory tract. There is growing evidence that the airway epithelium plays a direct role in mediating the immune response to foreign agents; therefore, characterizing the transcriptome of the airway epithelium in response to *A. fumigatus* conidia is an important step in understanding the full host immune response to *A. fumigatus*.

In the present study we have shown that primary AECs are capable of internalizing conidia of *A. fumigatus*. Several other studies have shown the uptake of conidia in other non-phagocytic cells including an alveolar type II cell line (A549) [13], primary tracheal epithelial cells, umbilical vein endothelial cells [14], and 16HBE14o- cells [17]. It has been previously suggested that epithelial cells may serve as reservoirs for fungal conidia during infection [12], [15], [16]. Alongside the uptake of *A. fumigatus* conidia by primary AECs, the current study identified 255 differentially expressed genes in 16HBE14o- cells exposed for 6 hours to conidia compared to non-exposed cells. The magnitude of the fold change tended to be modest, and is consistent with an earlier study by Zhang *et al.*, who found only minor changes in immune response effectors in the A549 cell line following exposure to conidia [31]. In addition, Aimanianda *et al.* [32] showed that the presence of hydrophobins on the conidial surface silenced the immune response, at least by dendritic cells and macrophages. Within microarray data it can often be difficult to identify any unifying signaling pathways or networks, particularly for instances in which the transcriptional response is only moderate. Although no single gene may drastically change in expression, a 20% change in members of a particular pathway may result in significant biological outcomes [33]. To identify whether a biologically relevant response was present, we employed GOEAST, GSEA, and IPA which enabled the identification of curated pathways significantly enriched within our dataset. Each of these tools independently identified gene sets involved in an innate immune response; chemokine activity, the defense response, and the inflammatory response among the most highly enriched annotations.

To help validate the physiological relevance of these results, we used RT-qPCR to evaluate selected genes derived from the most prevalent themes in the microarray set in both 16HBE14o- and primary AECs. The RT-qPCR data confirmed that the cytokine IL-6 was up-regulated upon exposure of either 16HBE14o- or AECs to the fungal conidia. Transgenic mouse strains deficient in IL-6 show an increased susceptibility to invasive pulmonary aspergillosis [34]. Cenci *et al.* [34] also found that IL-6 deficiency is associated with decreased antifungal effector functions of phagocytes and an impaired development of protective type 1 response. It has also been shown that the transformed Type II epithelial cell line, A549, secretes IL-6 in response to co-incubation with inactivated conidia [31], [35], [36]. However, to our knowledge, this is the first report of IL-6 expression by primary human AECs in response to co-incubation with the conidia of *A. fumigatus*. IL-6 is a potent pro-inflammatory cytokine, produced by a diverse set of cell populations, and exerts inflammatory effects by activating both leukocytes and structural cells including pulmonary epithelial cells. IL-6 is a part of the acute phase response, and is a potent inducer of C-reactive protein expression in the liver, which is important in systemic inflammation. IL-6 release has been demonstrated to be up-regulated during exacerbation periods of several respiratory diseases including asthma, cystic fibrosis and COPD [37]. It would be interesting to compare the relative expression of this cytokine by normal airway epithelia to airway epithelia derived from asthmatics following exposure to *A. fumigatus* conidia.

Other than IL-6, no replicable result between the responses of 16HBE14o- and AECs was observed for the human genes tested. CSF2 and MMP1 achieved significant differential expression in only one of the two cell types, failing to replicate in 16HBE14o- and AECs, respectively. This suggests fundamental differences in gene expression responses exist between the cell line and primary cells when they are exposed to conidia. This result underlines the necessity of replicating findings from cell lines in more physiologically-relevant model systems.

The 150 *A. fumigatus* genes up-regulated in response to interaction with 16HBE14o- cells in culture (compared to conidia incubated with media alone) were related to vacuolar acidification, siderophore biosynthesis, metallopeptidase and formate dehydrogenase activities. This value is comparable to the findings of Sugui *et al.*, who reported that 244 genes were up-regulated in conidia exposed to neutrophils [26]. In a comparison of our data with that of Sugui *et al.*, we identified 13 genes that overlapped between the two studies [26] (Table 3).

**Table 3:** List of *A. fumigatus* genes commonly identified by our study and by that of Sugui *et al*.

Locus ID	Gene name *(designation)*	P-value	Fold-change
Afu7g06770	hypothetical protein	2.79E-04	1.87
Afu3g08900	tubulin-specific chaperone c, putative	4.41E-04	2.67
Afu4g10410	aspartate aminotransferase, putative	2.38E-03	2.19
Afu6g03590	methylcitrate synthase	4.21E-03	2.06
Afu6g10260	aldehyde reductase (AKR1), putative	5.83E-03	1.90
Afu8g07130	antioxidant protein LsfA	1.60E-02	1.73
Afu4g08580	antioxidant protein LsfA	1.89E-02	−2.58
Afu3g10000	cAMP-dependent protein kinase regulatory subunit PkaR	2.62E-02	1.76
Afu4g12950	PX domain protein	2.74E-02	2.11
Afu2g11900	pyruvate dehydrogenase kinase	2.87E-02	2.45
Afu2g14590	MFS monosaccharide transporter, putative	3.05E-02	1.98
Afu3g00900	alpha-amylase AmyA	3.80E-02	1.62
Afu6g03730	prpd protein	4.08E-02	1.58

A general concordance was found between our microarray data and RT-qPCR data (Figure 3); of the eight *A. fumigatus* genes selected for validation with RT-qPCR, six remained significant following co-incubation with 16HBE14o- and five remained significant after incubation with primary AECs. *A. fumigatus* formate dehydrogenase (*fdh*) was significantly up-regulated. *Fdh* up-regulation was also observed by Sugui *et al.* [26] following exposure of *A. fumigatus* conidia to neutrophils. Up-regulation of *fdh* has been reported as a response of *Candida albicans* following exposure to human neutrophils, and is suspected of being involved in the detoxification of formate [38]. *Fdh* transcription has been shown to increase after 48 hours of fungal biofilm development, when compared to 24 hours [39]. Together, these data indicate that *fdh* up-regulation is an early response of fungi not only to professional phagocytes but also as a result of their interaction with epithelial cells.

The V-ATPase 98 kDa subunit was up-regulated in *A. fumigatus* incubated with AECs or 16HBE14o- cells. V-ATPases are structurally conserved proton pumps found in all eukaryotes that function to acidify the lumen of vacuoles [40]. In yeast, mutants lacking the V-ATPase (*vma* mutants) show enhanced sensitivity to oxidative stress, alkaline pH, metal ion stress, and high concentrations of non-fermentable carbon sources [40]. A similar phenotype has been observed in *Aspergillus nidulans*; a strain lacking the V-ATPase subunit was unable to grow in alkaline (>pH 7.0) conditions or high cation concentrations [41]. In fact, the importance of V-ATPase is highlighted by a recent study indicating that azoles mediate their toxicity by inhibiting ergosterol biosynthesis, which in turn results in the fatal dysregulation of V-ATPase function [42]. Although the utility of up-regulating vacuolar acidification mechanisms in the presence of human AECs is unknown, we hypothesize that V-ATPase up-regulation would promote cation uptake (such as mobilized free iron), modulate pH within the fungal cytosol, and/or assist in nutrient degradation (*e.g.*, stored conidial lipids) within the vacuole. A putative sulphur metabolism regulator gene, *skpA*, was also up-regulated in conidia following exposure to both AECs and 16HBE14o- cells. *SkpA* was shown to be up-regulated in mature biofilms of *A. fumigatus*, and may represent a requirement for methionine and cysteine-rich proteins during exposure to epithelial cells [39].

*MepB* is a metallopeptidase that has been shown to hydrolyze Type I collagen [43]. Mutants of *A. fumigatus* with a disruption in the *MepB* gene showed no phenotype in mouse models of invasive aspergillosis suggesting that this peptidase is not required for virulence [43]. In our study, we found no changes in *MepB* expression in conidia incubated with AECs in agreement with the *in vivo* results. In contrast, *SidA* catalyses the first committed step of hydroxamate-type siderophore biosysnthesis, and *SidA* has been found to be absolutely essential in *A. fumigatus* virulence [24], [25]. The ability of *A. fumigatus* to survive in serum is dependent on the removal of iron from host transferrin, indicating a role for *in vivo* siderophore biosynthesis [44]. We found an up-regulation of *SidA* suggesting that conidia face iron-limiting conditions when interacting with bronchial epithelium. These data are in agreement with the work of Schrettl *et al.* [45] who found that inhibiting siderophore biosynthesis reduced fungal growth rate in alveolar macrophages. This represents another example of fungal genes up-regulated in response to professional phagocytes that were also found to have increased expression with airway epithelial cells.

In conclusion, we have demonstrated the up-regulation of IL-6 by primary human AECs, along with several genes differentially regulated by conidia of *A. fumigatus* following their interaction with human epithelial cells. Interestingly, we found discrepancies in the transcriptional response of the 16HBE14o- cell line and primary human airway epithelial cells to *A. fumigatus* conidia. This suggests that some of the differences between primary cells and cell lines are relevant to interaction with *A. fumigatus* conidia, and caution should be exercised when choosing a model system. As we investigated only a subset of the differentially expressed genes of either species, further characterization may assist in identifying mechanisms of *A. fumigatus* pathogenesis. Future studies comparing the responses of epithelial cells from normal airways with those from individuals with diseased/abnormal airways (*e.g.*, cystic fibrosis, asthma) may contribute to understanding the characteristics that make individuals with underlying respiratory conditions vulnerable to *A. fumigatus* mediated diseases. Profiling temporal changes in the expression patterns of both species will also provide crucial insights into dynamic changes in expression anticipated to occur during the early phases of interaction.

## Supporting Information

Figure S1 **Summarized Ingenuity network used to generate RT-qPCR targets.** Pathway analysis of differentially expressed genes (purple) was used to select 5 human gene targets (green) for validation by RT-qPCR. The clustering of differentially expressed genes was used to triangulate nodes of interest, based on number and strength of direct and indirect connections. This was performed in an attempt to identify genes that are key drivers of the observed changes in expression, but may not have been identified in the microarray. In this way, we are allowing for downstream or co-regulated genes to implicate hubs in the network. These hubs of interest were then evaluated based on near-significance (data not shown) and chosen in rank order for validation.
(TIF)

Table S1 **TaqMan® gene expression assays used for the human RT-qPCR assays.**
(DOCX)

Table S2 **Oligonucleotides used for** ***A. fumigatus*** **RT-qPCR assays.**
(DOCX)

Table S3 **Human genes showing differential expression between 16HBE14o- cells incubated with and without conidia.**
(DOCX)

Table S4 **Fungal genes showing differential expression between conidia of** ***Aspergillus fumigatus*** **incubated with and without 16HBE14o- cells.**
(DOCX)
