# Supplementary figures and images for: Dual Organism Transcriptomics of Airway Epithelial Cells Interacting with Conidia of Aspergillus fumigatus
Source: PLoS One. 2011 May 31;6(5):e20527. doi: 10.1371/journal.pone.0020527 (PMC3105077; doi:10.1371/journal.pone.0020527)

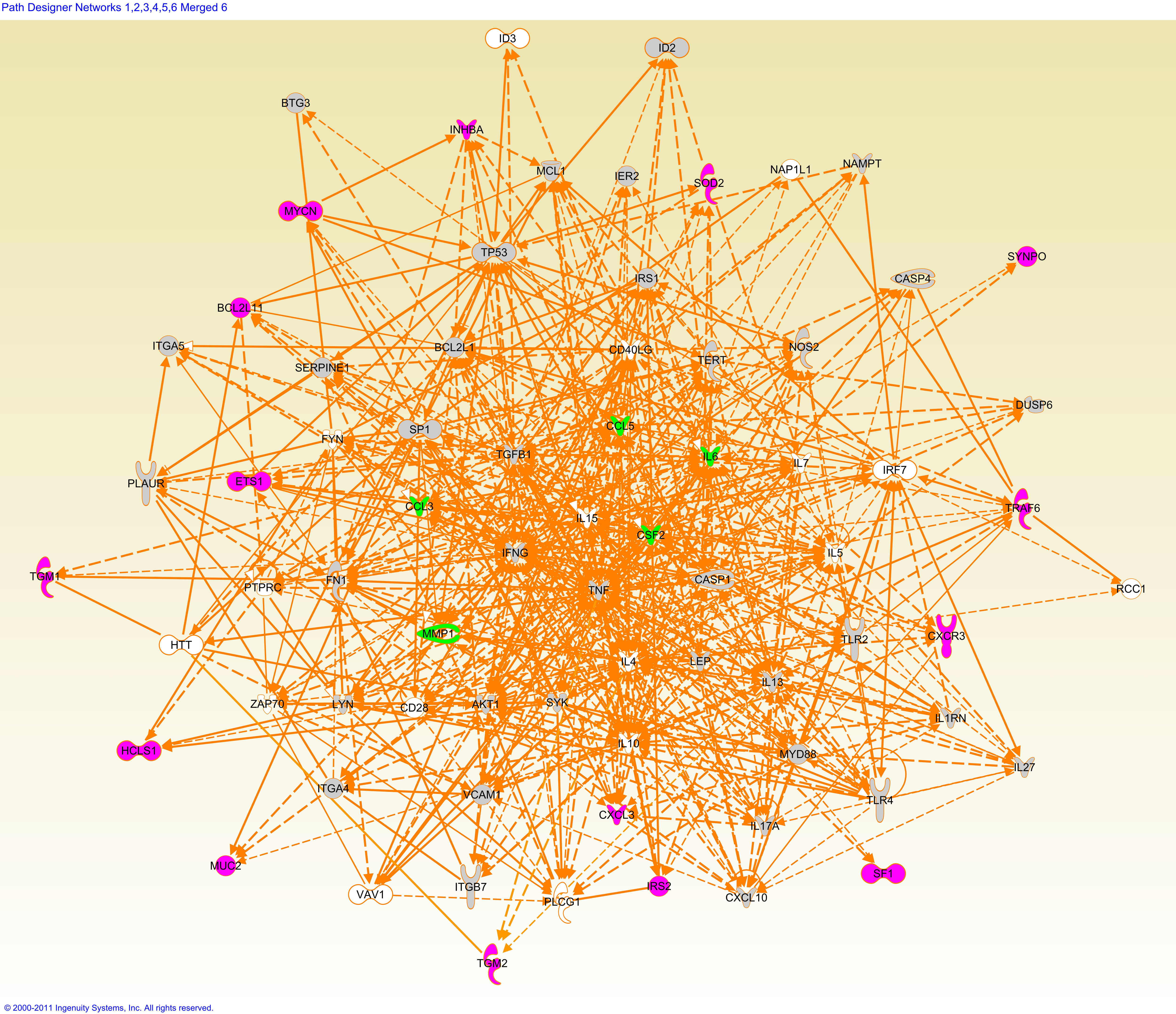

Supplement: Figure S1 — Summarized Ingenuity network used to generate RT-qPCR targets. Pathway analysis of differentially expressed genes (purple) was used to select 5 human gene targets (green) for validation by RT-qPCR. The clustering of differentially expressed genes was used to triangulate nodes of interest, based on number and strength of direct and indirect connections. This was performed in an attempt to identify genes that are key drivers of the observed changes in expression, but may not have been identified in the microarray. In this way, we are allowing for downstream or co-regulated genes to implicate hubs in the network. These hubs of interest were then evaluated based on near-significance (data not shown) and chosen in rank order for validation. (TIF) [file pone.0020527.s001.tif]
