# Supplementary material for: Dual Organism Transcriptomics of Airway Epithelial Cells Interacting with Conidia of Aspergillus fumigatus
Source: PLoS One. 2011 May 31;6(5):e20527. doi: 10.1371/journal.pone.0020527 (PMC3105077; doi:10.1371/journal.pone.0020527)
Supplement: Table S1 — TaqMan® gene expression assays used for the human RT-qPCR assays. (DOCX) [file pone.0020527.s002.docx]

**Table S1. TaqMan® gene expression assays used for the human RT-qPCR assays.**

| **Gene (designation)** | **ABI TaqMan kit** |
| --- | --- |
| human chemokine (C-C motif) ligand 3 (CCL3) | Hs00234142_m1 |
| human chemokine (C-C motif) ligand 5 (CCL5) | Hs00174575_m1 |
| human interleukin 6 (IL-6) | Hs00985641_m1 |
| human colony stimulating factor 2 (CSF2) | Hs00171266_m1 |
| human matrix metallopeptidase 1 (MMP1) | Hs00899658_m1 |
| human peptidylprolyl isomerase A (PPIA) | Hs99999904_m1 |
| human phosphoglycerate kinase 1 (PGK1) | Hs99999906_m1 |
| human zinc finger protein 433 (ZNF433) | Hs00908429_m1 |
| human leucine rich repeat containing 14 (LRRC14) | Hs00206378_m1 |
| human DOT1-like, histone H3 methyltransferase (DOT1L) | Hs00287200_m1 |
