# Supplementary material for: Dual Organism Transcriptomics of Airway Epithelial Cells Interacting with Conidia of Aspergillus fumigatus
Source: PLoS One. 2011 May 31;6(5):e20527. doi: 10.1371/journal.pone.0020527 (PMC3105077; doi:10.1371/journal.pone.0020527)
Supplement: Table S2 — Oligonucleotides used for A. fumigatus RT-qPCR assays. (DOCX) [file pone.0020527.s003.docx]

**Table S2. Oligonucleotides used for *A. fumigatus* RT-qPCR assays.**

| **Locus ID** | **Probe** | **Forward Primer** | **Reverse Primer** |
| --- | --- | --- | --- |
| Afu1g14710/beta-glucosidase | AGAGGGATGACACGAGACCATGAGAT | AGGATATTGCCCTGCTCAAG | CTTGACATAGTGCTGGAGACC |
| Afu7g05930/*MepB* | TGCAATTTCACCAAGCCCACGG | CCAACTTCAATCTCCAGCCG | AAGCTCATGGAACAGAGTCAC |
| Afu6g04920/NAD-dependent formate dehydrogenase | TGCTGTTTGGCGTGCTCACC | TACAGGGCAAGGTTCTTATGG | TCGATCCACTTTCTCAAGCC |
| Afu5g06060/*SkpA* | CGAGGTGGTGCTGAAGAAGGTCATT | GAACATGCTGGAAGATTTGGG | GGAGGGTCGTTCTTGTGATG |
| Afu2g02680/*MAP-1* | TTCATGGTGATGGACTCCGCGTT | GTGCTGACATTGCTAACTGTG | TGATTTCTTTTCCAAACCGCC |
| Afu3g08900/tubulin-specific chaperone C, putative | AGCCTCGCCCCAAATTTACCTTCA | CTATATCCCCACCTATGACCAAC | GCATCTGATAGCGAGACAGC |
| Afu4g11300/V-ATPase 98kDa subunit | CCAGCGGACTTTTACCAAGGAGATTCG | TCTTAACCCCGACACCAATG | ACGTATCGGAGAACTCGGTAG |
| Afu5g01970/*gpdA* | CCTTGATGGCCTGCTTGATCTCGT | CATGCGTGTTCCTACCTCC | CAGTGTAGCCGAGGATGTTC |
| Afu2g07680/*SidA* | CCGCATTGCAGAATCCCGCATAA | AAGAGATATCCCAACTCACGAAC | AGATTGGCTGTAGAACTTGTCG |
