# Supplementary material for: Dual Organism Transcriptomics of Airway Epithelial Cells Interacting with Conidia of Aspergillus fumigatus
Source: PLoS One. 2011 May 31;6(5):e20527. doi: 10.1371/journal.pone.0020527 (PMC3105077; doi:10.1371/journal.pone.0020527)
Supplement: Table S3 — Human genes showing differential expression between 16HBE14o- cells incubated with and without conidia. (DOCX) [file pone.0020527.s004.docx]

**Table S3. Human genes showing differential expression between 16HBE14o- cells incubated with and without conidia.** 255 genes were identified as showing differential expression based on a t-test (p-value cutoff of 0.05) and a fold change cutoff of 1.5. The p-values indicated are not adjusted for multiple testing. The genes were sorted by fold change.

| Probeset ID | Gene Name | P-value | Fold-change |
| --- | --- | --- | --- |
| A_24_P419028 | MOP-1 | 2.22E-02 | 2.98 |
| A_24_P348885 | CYB561D1 | 1.94E-02 | 2.15 |
| A_23_P39265 | LYPD3 | 1.18E-02 | 2.13 |
| A_24_P306034 | ANKDD1A | 4.40E-02 | 2.10 |
| A_23_P358370 | FOXE1 | 2.61E-02 | 2.06 |
| A_24_P15797 | DNAJC4 | 6.53E-03 | 2.04 |
| A_24_P483956 | A_24_P483956 | 8.17E-04 | 2.01 |
| A_24_P366122 | ACBD4 | 1.78E-02 | 2.01 |
| A_23_P82088 | NRN1 | 1.24E-02 | 2.00 |
| A_24_P252223 | C6orf85 | 1.66E-02 | 1.97 |
| A_24_P754989 | A_24_P754989 | 4.45E-02 | 1.93 |
| A_23_P259141 | ZBP1 | 5.56E-03 | 1.87 |
| A_24_P401270 | LOC649294 | 3.73E-02 | 1.86 |
| A_24_P752279 | A_24_P752279 | 2.71E-02 | 1.84 |
| A_24_P183150 | CXCL3 | 9.14E-03 | 1.83 |
| A_24_P480206 | A_24_P480206 | 1.97E-02 | 1.83 |
| A_32_P206175 | THC2429183 | 3.32E-02 | 1.81 |
| A_32_P156776 | AA360388 | 2.14E-02 | 1.80 |
| A_23_P380318 | EGR4 | 3.49E-03 | 1.78 |
| A_24_P76288 | A_24_P76288 | 2.14E-02 | 1.78 |
| A_24_P161144 | MGC46336 | 1.89E-02 | 1.78 |
| A_24_P230195 | A_24_P230195 | 1.50E-02 | 1.76 |
| A_23_P34554 | CACNA1E | 4.60E-02 | 1.76 |
| A_23_P208482 | CLEC4M | 1.99E-02 | 1.75 |
| A_23_P78952 | PIP5K1C | 1.15E-02 | 1.74 |
| A_23_P136753 | THC2275252 | 2.91E-02 | 1.74 |
| A_24_P375586 | A_24_P375586 | 3.50E-02 | 1.73 |
| A_24_P930551 | CV399637 | 5.33E-03 | 1.72 |
| A_23_P122924 | INHBA | 1.26E-03 | 1.72 |
| A_23_P118370 | AK022252 | 1.77E-03 | 1.71 |
| A_24_P256155 | NKX1-2 | 2.02E-02 | 1.71 |
| A_23_P54692 | FLJ12547 | 7.56E-03 | 1.71 |
| A_24_P331711 | THEM5 | 1.59E-02 | 1.71 |
| A_24_P166434 | PSORS1C2 | 1.04E-02 | 1.70 |
| A_23_P136413 | MMP17 | 1.90E-02 | 1.70 |
| A_23_P17030 | RNPEPL1 | 2.66E-03 | 1.69 |
| A_24_P631848 | BC036435 | 1.34E-02 | 1.69 |
| A_24_P269101 | NEUROG1 | 2.12E-02 | 1.68 |
| A_24_P24244 | ATN1 | 1.26E-02 | 1.68 |
| A_23_P53838 | IRS2 | 1.41E-02 | 1.68 |
| A_23_P23815 | SLC30A1 | 2.65E-03 | 1.68 |
| A_24_P770494 | A_24_P770494 | 1.22E-02 | 1.67 |
| A_24_P478362 | THC2367544 | 3.54E-02 | 1.67 |
| A_24_P322088 | CR619805 | 2.42E-02 | 1.67 |
| A_24_P922101 | THC2269657 | 8.58E-03 | 1.67 |
| A_24_P65292 | SOX8 | 3.21E-02 | 1.67 |
| A_32_P74409 | ENST00000339446 | 3.34E-03 | 1.66 |
| A_24_P316454 | BC022826 | 2.95E-02 | 1.66 |
| A_32_P145764 | BC043547 | 2.84E-03 | 1.65 |
| A_24_P401294 | FLJ35934 | 2.44E-02 | 1.65 |
| A_24_P647965 | A_24_P647965 | 4.86E-02 | 1.65 |
| A_24_P20795 | IRX4 | 1.66E-02 | 1.65 |
| A_32_P116219 | A_32_P116219 | 2.72E-02 | 1.64 |
| A_24_P310009 | USP43 | 1.30E-02 | 1.64 |
| A_24_P247920 | KIAA1652 | 1.60E-02 | 1.63 |
| A_24_P180830 | CARD9 | 2.25E-02 | 1.62 |
| A_24_P828125 | A_24_P828125 | 2.53E-02 | 1.62 |
| A_23_P258381 | SPSB4 | 3.31E-02 | 1.62 |
| A_24_P937095 | ENST00000367001 | 7.98E-04 | 1.62 |
| A_24_P935819 | SOD2 | 3.23E-02 | 1.61 |
| A_23_P372962 | A2ML1 | 1.37E-02 | 1.61 |
| A_23_P73429 | HCLS1 | 4.68E-02 | 1.60 |
| A_24_P264383 | THC2251316 | 3.46E-02 | 1.59 |
| A_32_P199506 | A_32_P199506 | 1.28E-02 | 1.59 |
| A_24_P315581 | A_24_P315581 | 3.37E-02 | 1.59 |
| A_24_P281025 | A_24_P281025 | 1.56E-02 | 1.59 |
| A_23_P140527 | FOXB1 | 9.27E-03 | 1.59 |
| A_23_P329212 | ETS1 | 2.72E-05 | 1.59 |
| A_32_P160254 | DKFZP434I0714 | 1.84E-02 | 1.58 |
| A_32_P39866 | BC040412 | 3.08E-02 | 1.58 |
| A_24_P778928 | THC2410279 | 3.79E-02 | 1.58 |
| A_24_P127543 | A_24_P127543 | 4.03E-02 | 1.58 |
| A_23_P114299 | CXCR3 | 2.57E-03 | 1.57 |
| A_24_P127425 | A_24_P127425 | 1.50E-02 | 1.57 |
| A_24_P247408 | LOC399851 | 4.53E-02 | 1.57 |
| A_23_P201156 | IGSF4B | 4.68E-03 | 1.56 |
| A_32_P122907 | THC2440913 | 3.29E-02 | 1.56 |
| A_23_P4400 | KRTAP4-14 | 2.77E-02 | 1.56 |
| A_24_P329353 | TMEM142B | 5.67E-03 | 1.56 |
| A_24_P368943 | EVX1 | 1.10E-02 | 1.55 |
| A_23_P90339 | SF3A2 | 1.55E-02 | 1.54 |
| A_23_P303390 | MYCN | 4.64E-03 | 1.54 |
| A_24_P213494 | PTPRE | 3.78E-02 | 1.54 |
| A_24_P689119 | A_24_P689119 | 2.66E-02 | 1.54 |
| A_24_P143032 | CDC42EP1 | 1.32E-02 | 1.54 |
| A_32_P194115 | UNC84B | 3.89E-03 | 1.54 |
| A_24_P8130 | AK092421 | 1.78E-02 | 1.54 |
| A_24_P322771 | TFF1 | 4.01E-02 | 1.53 |
| A_24_P450870 | THC2334619 | 4.76E-03 | 1.53 |
| A_32_P234543 | A_32_P234543 | 2.96E-03 | 1.53 |
| A_23_P309720 | GABRD | 2.37E-03 | 1.53 |
| A_32_P158253 | A_32_P158253 | 3.13E-02 | 1.53 |
| A_23_P389426 | MGC26597 | 1.64E-03 | 1.52 |
| A_32_P77977 | ENST00000373014 | 8.41E-04 | 1.52 |
| A_32_P6769 | D2HGDH | 4.48E-02 | 1.52 |
| A_24_P315066 | DKFZp761I2123 | 1.59E-02 | 1.52 |
| A_23_P119395 | GIPR | 4.74E-03 | 1.52 |
| A_24_P395966 | ZBP1 | 2.12E-03 | 1.52 |
| A_24_P405992 | SYNPO | 3.63E-02 | 1.51 |
| A_32_P1516 | AA714537 | 2.01E-02 | 1.51 |
| A_24_P207003 | IRS2 | 6.33E-03 | 1.51 |
| A_24_P848662 | CR594528 | 2.87E-02 | 1.51 |
| A_23_P26468 | RHBDL1 | 4.66E-02 | 1.51 |
| A_32_P106925 | THC2356891 | 2.86E-02 | 1.51 |
| A_32_P8724 | THC2409364 | 2.08E-02 | 1.51 |
| A_24_P341761 | MUC2 | 7.39E-03 | 1.50 |
| A_23_P250206 | GCK | 6.97E-03 | 1.50 |
| A_32_P76566 | THC2392192 | 1.66E-02 | 1.50 |
| A_24_P280497 | KIAA1545 | 3.90E-02 | 1.50 |
| A_23_P150841 | ZNF140 | 2.60E-02 | -1.50 |
| A_23_P127522 | HYLS1 | 1.65E-02 | -1.50 |
| A_24_P91405 | ZNF709 | 3.06E-03 | -1.50 |
| A_23_P23102 | ZNF31 | 1.16E-02 | -1.51 |
| A_23_P218282 | ZNF434 | 3.45E-04 | -1.51 |
| A_24_P665185 | GPR137B | 1.89E-02 | -1.51 |
| A_24_P82200 | MEIS2 | 1.96E-03 | -1.51 |
| A_23_P126197 | SFRS4 | 1.11E-03 | -1.51 |
| A_23_P87973 | RFP2 | 3.36E-02 | -1.51 |
| A_23_P59855 | ZNF138 | 3.91E-04 | -1.51 |
| A_23_P8004 | ENST00000356177 | 4.08E-02 | -1.51 |
| A_24_P238578 | KIAA1729 | 4.61E-02 | -1.51 |
| A_24_P24263 | PDLIM5 | 7.24E-03 | -1.51 |
| A_23_P163047 | WDR89 | 2.21E-04 | -1.51 |
| A_32_P174398 | AF085351 | 1.78E-03 | -1.51 |
| A_24_P234196 | RRM2 | 1.66E-04 | -1.51 |
| A_23_P101351 | ZNF426 | 1.48E-04 | -1.52 |
| A_23_P320190 | ENST00000272831 | 6.48E-04 | -1.52 |
| A_32_P23010 | ENST00000378887 | 5.55E-03 | -1.52 |
| A_23_P408768 | DOT1L | 2.98E-02 | -1.52 |
| A_23_P80839 | MAP6D1 | 6.87E-04 | -1.52 |
| A_32_P18073 | BF812215 | 1.75E-03 | -1.52 |
| A_23_P208325 | ZNF235 | 4.40E-03 | -1.53 |
| A_23_P55911 | AK092559 | 2.73E-02 | -1.53 |
| A_23_P84836 | NPEPPS | 1.20E-02 | -1.53 |
| A_23_P61268 | C8orf30A | 2.04E-03 | -1.53 |
| A_23_P70794 | RAB23 | 1.56E-03 | -1.53 |
| A_24_P198629 | LINS1 | 2.26E-02 | -1.53 |
| A_24_P61520 | MLH3 | 7.69E-04 | -1.53 |
| A_23_P155027 | MORC2 | 3.34E-02 | -1.53 |
| A_24_P169544 | ZNF17 | 3.93E-02 | -1.53 |
| A_23_P80940 | PPAT | 2.10E-04 | -1.53 |
| A_23_P353106 | FAM105B | 1.14E-02 | -1.53 |
| A_23_P32249 | ENST00000372415 | 1.26E-02 | -1.53 |
| A_32_P24709 | ZNF642 | 1.03E-02 | -1.53 |
| A_24_P532212 | AK026896 | 1.06E-02 | -1.53 |
| A_23_P320407 | AF318318 | 8.27E-03 | -1.53 |
| A_23_P108342 | ZNF571 | 3.55E-03 | -1.53 |
| A_23_P311640 | HRBL | 5.42E-03 | -1.54 |
| A_24_P917026 | NF1 | 7.06E-04 | -1.54 |
| A_23_P91350 | ENST00000379019 | 4.35E-02 | -1.54 |
| A_23_P104138 | MGC15634 | 7.89E-03 | -1.55 |
| A_23_P83134 | GAS1 | 7.28E-03 | -1.55 |
| A_23_P120170 | TIGD1 | 2.61E-03 | -1.55 |
| A_24_P466102 | NP1167346 | 2.34E-03 | -1.55 |
| A_24_P274615 | ARRDC3 | 4.81E-03 | -1.55 |
| A_24_P376129 | DFNB31 | 1.96E-02 | -1.55 |
| A_23_P405942 | LARP5 | 1.20E-02 | -1.55 |
| A_23_P20683 | KIAA0020 | 9.48E-04 | -1.55 |
| A_32_P24651 | AK095707 | 9.34E-04 | -1.55 |
| A_24_P414719 | FLJ11236 | 9.56E-03 | -1.56 |
| A_24_P98613 | TSPAN14 | 1.29E-02 | -1.56 |
| A_32_P78783 | FLJ31875 | 2.15E-03 | -1.56 |
| A_23_P258037 | JMJD1A | 6.38E-03 | -1.56 |
| A_23_P39263 | ZNF57 | 3.84E-02 | -1.56 |
| A_23_P18384 | ARMC8 | 3.08E-02 | -1.56 |
| A_24_P51118 | MTAP | 1.23E-02 | -1.56 |
| A_23_P402287 | LNX2 | 2.56E-02 | -1.57 |
| A_32_P219942 | THC2374204 | 1.66E-05 | -1.57 |
| A_23_P322 | EFNA4 | 3.08E-03 | -1.57 |
| A_24_P419276 | ZNF248 | 2.51E-02 | -1.57 |
| A_23_P216476 | ZBTB5 | 3.79E-03 | -1.57 |
| A_23_P92184 | WDR5B | 6.88E-03 | -1.58 |
| A_24_P268893 | THAP6 | 3.27E-03 | -1.58 |
| A_23_P385246 | KCTD6 | 5.83E-03 | -1.58 |
| A_23_P205265 | EIF5 | 3.23E-03 | -1.58 |
| A_23_P128375 | C12orf34 | 4.02E-02 | -1.58 |
| A_23_P155939 | ZNF595 | 5.09E-03 | -1.58 |
| A_24_P198820 | THC2435239 | 9.96E-03 | -1.58 |
| A_24_P491087 | LOC441244 | 4.66E-02 | -1.59 |
| A_23_P405707 | BCOR | 3.00E-05 | -1.59 |
| A_24_P177585 | FLJ40869 | 2.88E-03 | -1.60 |
| A_24_P923251 | TGM2 | 7.48E-03 | -1.60 |
| A_23_P161686 | RICS | 5.57E-04 | -1.60 |
| A_24_P344537 | ZNF625 | 1.11E-03 | -1.60 |
| A_24_P931636 | TBRG1 | 3.03E-03 | -1.60 |
| A_24_P178065 | PHLDB2 | 3.39E-02 | -1.61 |
| A_32_P51119 | STOX1 | 5.75E-03 | -1.61 |
| A_24_P934679 | THC2426708 | 5.17E-03 | -1.61 |
| A_23_P2032 | A_23_P2032 | 2.67E-03 | -1.61 |
| A_23_P146325 | DDEF1IT1 | 2.64E-03 | -1.61 |
| A_23_P108437 | C2orf31 | 5.64E-04 | -1.62 |
| A_23_P80062 | TAF4 | 1.99E-04 | -1.62 |
| A_24_P22976 | ARRDC2 | 9.66E-03 | -1.63 |
| A_23_P44505 | KLF11 | 1.40E-03 | -1.63 |
| A_24_P167614 | INTS6 | 3.83E-02 | -1.63 |
| A_23_P65797 | KLHL25 | 4.09E-02 | -1.63 |
| A_23_P130965 | ARRDC2 | 3.71E-03 | -1.64 |
| A_32_P145385 | AK001118 | 8.40E-03 | -1.64 |
| A_32_P229818 | AK022044 | 6.53E-03 | -1.64 |
| A_23_P131935 | C20orf42 | 5.03E-03 | -1.65 |
| A_24_P414446 | FAM105B | 3.63E-03 | -1.65 |
| A_24_P235783 | SF1 | 8.10E-03 | -1.65 |
| A_24_P917015 | DYNC2H1 | 1.26E-02 | -1.67 |
| A_23_P309996 | BCL2L11 | 5.68E-03 | -1.67 |
| A_24_P48057 | IRX5 | 4.61E-03 | -1.67 |
| A_23_P135730 | ZNF627 | 7.27E-04 | -1.67 |
| A_23_P65618 | TGM1 | 2.24E-03 | -1.67 |
| A_23_P356139 | C10orf6 | 3.04E-03 | -1.68 |
| A_23_P115861 | ZNF485 | 3.70E-03 | -1.68 |
| A_23_P75921 | TRAF6 | 1.12E-02 | -1.69 |
| A_23_P206741 | A_23_P206741 | 5.73E-03 | -1.69 |
| A_24_P491923 | THC2273623 | 8.02E-03 | -1.69 |
| A_23_P7301 | WHSC1 | 1.38E-03 | -1.69 |
| A_24_P105761 | JMJD1A | 1.10E-03 | -1.69 |
| A_23_P55880 | ZNF564 | 7.55E-04 | -1.70 |
| A_23_P219045 | HIST1H3D | 8.06E-03 | -1.70 |
| A_23_P134946 | LRRC14 | 7.43E-04 | -1.71 |
| A_32_P19917 | BM684461 | 5.59E-03 | -1.71 |
| A_24_P281243 | LOC389072 | 3.80E-02 | -1.72 |
| A_23_P85703 | SOX13 | 5.49E-04 | -1.72 |
| A_23_P93258 | HIST1H3B | 4.52E-03 | -1.72 |
| A_24_P937546 | CMTM7 | 3.34E-02 | -1.72 |
| A_23_P116602 | USP35 | 2.06E-03 | -1.73 |
| A_23_P27649 | ZNF433 | 5.20E-03 | -1.73 |
| A_32_P62371 | THC2391454 | 8.55E-03 | -1.73 |
| A_23_P39050 | ENST00000341191 | 2.21E-02 | -1.73 |
| A_24_P717586 | A_24_P717586 | 2.03E-03 | -1.74 |
| A_24_P173234 | ZNF613 | 1.57E-03 | -1.75 |
| A_23_P27638 | ZNF700 | 7.62E-04 | -1.77 |
| A_24_P329795 | C10orf10 | 2.98E-03 | -1.78 |
| A_23_P104318 | DDIT4 | 6.35E-04 | -1.79 |
| A_32_P105940 | A_32_P105940 | 5.66E-03 | -1.79 |
| A_32_P105110 | AK057196 | 1.36E-03 | -1.79 |
| A_23_P214425 | ENST00000341376 | 3.65E-03 | -1.80 |
| A_23_P67278 | ZNF443 | 4.44E-03 | -1.80 |
| A_23_P101476 | ZNF442 | 1.83E-02 | -1.80 |
| A_32_P184039 | A_32_P184039 | 3.84E-02 | -1.81 |
| A_23_P54447 | C15orf5 | 9.85E-03 | -1.81 |
| A_23_P106412 | AK130644 | 9.90E-03 | -1.83 |
| A_32_P208039 | THC2314205 | 4.57E-04 | -1.83 |
| A_23_P27636 | ZNF700 | 2.75E-04 | -1.84 |
| A_24_P128255 | THC2275950 | 3.29E-03 | -1.84 |
| A_23_P379746 | MGC24039 | 1.51E-03 | -1.87 |
| A_32_P109296 | C15orf42 | 3.91E-04 | -1.87 |
| A_23_P333484 | HIST1H3H | 7.90E-03 | -1.87 |
| A_32_P160670 | THC2341080 | 1.27E-03 | -1.93 |
| A_23_P52885 | C11orf61 | 3.09E-05 | -1.95 |
| A_23_P87532 | C1QDC1 | 4.88E-05 | -1.95 |
| A_23_P128060 | ZNF26 | 4.43E-02 | -2.00 |
| A_23_P345707 | C15orf42 | 7.14E-04 | -2.02 |
| A_24_P548881 | THC2281244 | 4.22E-02 | -2.03 |
| A_24_P65941 | C21orf96 | 2.03E-05 | -2.34 |
| A_23_P395426 | DIDO1 | 9.16E-03 | -2.61 |
| A_24_P42308 | AK056449 | 3.61E-03 | -3.16 |
| A_23_P30805 | HIST1H4J | 3.02E-04 | -4.13 |
