# Supplementary material for: Dual Organism Transcriptomics of Airway Epithelial Cells Interacting with Conidia of Aspergillus fumigatus
Source: PLoS One. 2011 May 31;6(5):e20527. doi: 10.1371/journal.pone.0020527 (PMC3105077; doi:10.1371/journal.pone.0020527)
Supplement: Table S4 — Fungal genes showing differential expression between conidia of Aspergillus fumigatus incubated with and without 16HBE14o- cells. (DOCX) [file pone.0020527.s005.docx]

**Table S4. Fungal genes showing differential expression between conidia of *Aspergillus fumigatus* incubated with and without 16HBE14o- cells.** 183 genes were identified as showing differential expression based on a t-test (p-value cutoff of 0.05) and a fold change cutoff of 1.5. The p-values indicated are not adjusted for multiple testing. The genes were sorted by fold change.

| Locus | Common Name of Primary Target | P-value | Fold-change |
| --- | --- | --- | --- |
| Afu2g14210 | mitochondrial dihydroxy acid dehydratase, putative | 3.47E-03 | 3.85 |
| Afu5g02370 | Vacuolar ATP synthase catalytic subunit A, putative | 1.13E-02 | 3.58 |
| Afu8g07210 | hydroxymethylglutaryl-CoA synthase | 6.82E-03 | 3.13 |
| Afu4g00270 | transposase | 2.27E-02 | 3.09 |
| Afu4g09030 | aminopeptidase | 1.38E-02 | 2.89 |
| Afu3g03940 | 2,3-diketo-5-methylthio-1-phosphopentane phosphatase, putative | 4.87E-02 | 2.79 |
| Afu3g08010 | C2H2 transcription factor (Ace1), putative | 1.64E-02 | 2.74 |
| Afu4g00660 | sensor histidine kinase/response regulator, putative | 4.24E-02 | 2.72 |
| Afu3g08900 | tubulin-specific chaperone c, putative | 4.41E-04 | 2.67 |
| Afu1g14710 | Beta-glucosidase | 8.35E-04 | 2.66 |
| Afu3g06610 | proteasome regulatory particle subunit (RpnE), putative | 2.20E-02 | 2.58 |
| Afu4g11530 | intermembrane space AAA protease IAP-1 | 2.79E-03 | 2.58 |
| Afu2g02470 | PA domain protein | 2.64E-02 | 2.54 |
| Afu2g02680 | matrix AAA protease MAP-1 | 3.42E-02 | 2.53 |
| Afu5g07860 | phosphatase family protein | 2.01E-02 | 2.52 |
| Afu5g14390 | C6 transcription factor, putative | 3.80E-02 | 2.51 |
| Afu2g07370 | hypothetical protein | 9.27E-03 | 2.50 |
| Afu4g11300 | vacuolar ATPase 98 kDa subunit, putative | 1.39E-02 | 2.50 |
| Afu2g11900 | pyruvate dehydrogenase kinase | 2.87E-02 | 2.45 |
| Afu3g12430 | guanine nucleotide exchange factor, putative | 3.72E-02 | 2.44 |
| Afu7g05930 | metallopeptidase MepB | 1.05E-03 | 2.42 |
| Afu8g04800 | valyl-tRNA synthetase | 1.18E-02 | 2.42 |
| Afu2g04820 | translation release factor eRF3, putative | 5.58E-03 | 2.41 |
| Afu3g12530 | sensor histidine kinase/response regulator, putative | 3.58E-02 | 2.41 |
| Afu5g10660 | pentatricopeptide repeat protein | 5.11E-03 | 2.41 |
| Afu2g07680 | L-ornithine N5-oxygenase | 1.70E-02 | 2.38 |
| Afu8g07350 | conserved hypothetical protein | 2.45E-02 | 2.36 |
| Afu3g06280 | Rho GTPase activator (Rgd1), putative | 1.95E-02 | 2.33 |
| Afu5g03560 | glutamyl-tRNA synthetase | 1.55E-02 | 2.31 |
| Afu3g12330 | phosphatidyl synthase | 9.70E-03 | 2.25 |
| Afu4g11840 | glyoxylate reductase | 1.46E-02 | 2.25 |
| Afu3g03560 | coenzyme A disulfide reductase | 2.27E-02 | 2.25 |
| Afu6g12170 | FKBP-type peptidyl-prolyl isomerase, putative | 3.53E-04 | 2.24 |
| Afu2g13680 | calcium/calmodulin-dependent protein kinase, putative | 2.63E-02 | 2.24 |
| Afu2g03490 | calcium/calmodulin-dependent protein kinase, putative | 3.38E-02 | 2.20 |
| Afu2g01450 | alpha-1,6 mannosyltransferase subunit (Mnn9), putative | 2.68E-02 | 2.19 |
| Afu4g10410 | aspartate aminotransferase, putative | 2.38E-03 | 2.19 |
| Afu2g03590 | Ribosomal protein S21e | 5.66E-04 | 2.17 |
| Afu2g07500 | prolidase pepP, putative | 3.16E-04 | 2.15 |
| Afu2g13630 | amino transferase | 1.33E-02 | 2.15 |
| Afu6g08050 | 6-phosphogluconate dehydrogenase, decarboxylating | 5.39E-04 | 2.14 |
| Afu3g07850 | dipeptidyl aminopeptidase (Ste13), putative | 1.20E-02 | 2.12 |
| Afu4g12950 | PX domain protein | 2.74E-02 | 2.11 |
| Afu7g01730 | phosphatidylserine decarboxylase family protein | 1.63E-03 | 2.10 |
| Afu7g05470 | electron transfer flavoprotein alpha subunit, putative | 1.43E-03 | 2.07 |
| Afu2g00310 | Transmembrane amino acid transporter protein family | 1.69E-02 | 2.07 |
| Afu6g03590 | methylcitrate synthase | 4.21E-03 | 2.06 |
| Afu6g13160 | serine/threonine protein kinase, putative | 3.18E-02 | 2.05 |
| Afu4g11340 | saccharopine dehydrogenase | 1.02E-03 | 2.04 |
| Afu3g04210 | fatty acid synthase alpha subunit, putative | 4.41E-02 | 2.03 |
| Afu3g10840 | zinc knuckle transcription factor (Zfm1), putative | 4.36E-02 | 2.02 |
| Afu5g00650 | hypothetical protein | 1.73E-02 | 2.01 |
| Afu4g11240 | alpha-aminoadipate reductase large subunit, putative | 3.43E-02 | 1.99 |
| Afu2g14590 | MFS monosaccharide transporter, putative | 3.05E-02 | 1.98 |
| Afu2g03120 | cell wall glucanase (Utr2), putative | 8.87E-03 | 1.97 |
| Afu2g10030 | vip1 protein | 2.67E-02 | 1.96 |
| Afu6g11260 | ribosomal protein L26 | 1.08E-04 | 1.96 |
| Afu3g12480 | hymA | 3.75E-03 | 1.95 |
| Afu4g12340 | mitochondrial carrier protein, putative | 1.34E-02 | 1.95 |
| Afu2g12260 | cytochrome c oxidase assembly protein cox11 | 1.33E-02 | 1.95 |
| Afu3g11100 | conserved hypothetical protein | 4.11E-02 | 1.93 |
| Afu2g03610 | IMP dehydrogenase, putative | 4.88E-02 | 1.92 |
| Afu2g02170 | nuclear condensin complex subunit Smc4, putative | 4.94E-02 | 1.92 |
| Afu5g06360 | 60s ribosomal protein yl6 | 6.64E-05 | 1.91 |
| Afu4g08970 | PAP2 domain protein | 1.61E-02 | 1.91 |
| Afu5g01960 | conserved hypothetical protein | 4.92E-03 | 1.90 |
| Afu6g10260 | aldehyde reductase (AKR1), putative | 5.83E-03 | 1.90 |
| Afu3g13380 | ribose-phosphate pyrophosphokinase | 1.52E-02 | 1.90 |
| Afu7g05840 | Amidohydrolase family superfamily | 5.84E-03 | 1.89 |
| Afu7g04580 | TBC domain protein, putative | 2.83E-02 | 1.89 |
| Afu8g04340 | cystathionine gamma-lyase | 2.44E-03 | 1.89 |
| Afu2g04990 | transposase | 1.86E-03 | 1.89 |
| Afu5g12990 | Snf1 protein kinase complex subunit Snf4, putative | 2.03E-02 | 1.89 |
| Afu3g12290 | Dim1p | 1.07E-02 | 1.88 |
| Afu5g05830 | CorA family metal ion transporter, putative | 4.17E-02 | 1.88 |
| Afu7g06770 | hypothetical protein | 2.79E-04 | 1.87 |
| Afu8g05580 | coenzyme A transferase PsecoA | 5.36E-03 | 1.86 |
| Afu4g05830 | IDI2 | 1.70E-02 | 1.86 |
| Afu8g01670 | bifunctional catalase-peroxidase Cat2 | 3.19E-03 | 1.85 |
| Afu7g01860 | heat shock protein (Sti1), putative | 3.51E-02 | 1.84 |
| Afu4g03930 | cysteine synthase B, putative | 3.45E-02 | 1.84 |
| Afu5g07050 | proteasome regulatory particle subunit Rpt2, putative | 8.41E-03 | 1.84 |
| Afu5g06660 | UPF0187 domain membrane protein | 4.23E-02 | 1.83 |
| Afu7g02310 | adenine phosphoribosyltransferase 1 | 8.74E-03 | 1.83 |
| Afu2g13240 | V-type ATPase, B subunit, putative | 4.43E-02 | 1.82 |
| Afu8g05260 | nucleic acid-binding protein | 4.14E-02 | 1.82 |
| Afu4g12000 | phosphatidylinositol phospholipase C | 2.07E-02 | 1.82 |
| Afu6g02230 | glucokinase GlkA, putative | 1.38E-02 | 1.82 |
| Afu6g00100 | transposase | 1.44E-02 | 1.81 |
| Afu6g06780 | proteasome regulatory particle subunit Rpt4, putative | 2.00E-02 | 1.81 |
| Afu8g05810 | DUF1295 domain protein | 1.22E-02 | 1.78 |
| Afu6g04570 | elongation factor 1-gamma 2 | 3.31E-02 | 1.78 |
| Afu6g06570 | conserved hypothetical protein | 3.82E-02 | 1.78 |
| Afu6g10380 | cullin binding protein CanA, putative | 6.45E-03 | 1.78 |
| Afu5g05550 | class V myosin (Myo4), putative | 4.09E-02 | 1.77 |
| Afu5g08930 | isovaleryl-CoA dehydrogenase | 1.46E-04 | 1.77 |
| Afu3g10000 | cAMP-dependent protein kinase regulatory subunit PkaR | 2.62E-02 | 1.76 |
| Afu7g04080 | 3-ketoacyl-CoA thiolase (POT1), putative | 2.84E-02 | 1.75 |
| Afu4g13180 | TPR repeat protein | 2.89E-02 | 1.74 |
| Afu3g09290 | phosphoglycerate mutase, 2,3-bisphosphoglycerate-independent | 4.73E-03 | 1.74 |
| Afu2g15770 | conserved hypothetical protein | 5.73E-03 | 1.74 |
| Afu2g12870 | vesicular-fusion protein sec17 | 3.86E-02 | 1.73 |
| Afu2g10870 | hypothetical protein | 4.17E-02 | 1.73 |
| Afu8g07130 | antioxidant protein LsfA | 1.60E-02 | 1.73 |
| Afu5g02470 | thiamine biosynthesis protein (Nmt1), putative | 2.41E-02 | 1.72 |
| Afu5g13450 | triosephosphate isomerase | 3.77E-05 | 1.72 |
| Afu2g00970 | alcohol dehydrogenase, zinc-containing | 2.94E-03 | 1.72 |
| Afu2g10220 | glycerol dehydrogenase, putative | 2.65E-02 | 1.72 |
| Afu2g05790 | oligosaccharyl transferase subunit (alpha), putative | 2.43E-03 | 1.70 |
| Afu7g04250 | protein-ER retention protein (Erd1), putative | 3.98E-02 | 1.70 |
| Afu6g06770 | enolase | 3.32E-03 | 1.67 |
| Afu6g06440 | proteasome component Prs3, putative | 1.26E-02 | 1.67 |
| Afu3g04300 | hypothetical protein | 1.99E-03 | 1.65 |
| Afu3g06970 | cytosolic small ribosomal subunit S9, putative | 3.51E-03 | 1.65 |
| Afu2g07620 | cystathionine beta-synthase, putative | 4.58E-02 | 1.63 |
| Afu5g02180 | cysteine synthase (o-acetylserine (thiol)-lyase) | 4.94E-02 | 1.63 |
| Afu8g02850 | actin binding protein, putative | 8.69E-03 | 1.63 |
| Afu2g01210 | ATP dependent RNA helicase (Dbp5), putative | 3.27E-02 | 1.63 |
| Afu3g00900 | alpha-amylase AmyA | 3.80E-02 | 1.62 |
| Afu4g13140 | hypothetical protein | 3.11E-03 | 1.62 |
| Afu7g03750 | serine/threonine protein kinase, putative | 1.32E-02 | 1.61 |
| Afu3g05370 | 2-oxoglutarate dehydrogenase, E2 component, dihydrolipoamide succinyltransferase | 3.93E-02 | 1.61 |
| Afu8g04920 | LEA domain protein | 8.61E-03 | 1.61 |
| Afu5g03140 | GTP cyclohydrolase i | 3.66E-02 | 1.60 |
| Afu5g05460 | cytosine deaminase-uracil phosphoribosyltransferase fusion protein | 4.34E-02 | 1.59 |
| Afu4g13120 | glutamine synthetase | 3.71E-02 | 1.59 |
| Afu3g14270 | aldo-keto reductase (AKR), putative | 1.26E-02 | 1.59 |
| Afu5g14330 | CPRD8 protein | 4.50E-02 | 1.58 |
| Afu6g03730 | prpd protein | 4.08E-02 | 1.58 |
| Afu6g02440 | 60s ribosomal protein L24, putative | 1.29E-03 | 1.58 |
| Afu7g02580 | transposase | 1.45E-02 | 1.57 |
| Afu6g04110 | CLPTM1 domain protein | 3.12E-02 | 1.57 |
| Afu5g04230 | citrate synthase, eukaryotic | 1.95E-02 | 1.56 |
| Afu3g07290 | SD08430p | 1.40E-02 | 1.56 |
| Afu6g07910 | PH domain protein | 1.18E-02 | 1.56 |
| Afu5g04290 | WW domain protein, putative | 2.49E-02 | 1.56 |
| Afu4g11730 | glycerol dehydrogenase (GldB), putative | 6.64E-03 | 1.55 |
| Afu6g04540 | calcineurin regulatory subunit | 3.85E-02 | 1.55 |
| Afu6g13250 | Ribosomal protein L31e | 2.68E-03 | 1.55 |
| Afu2g04220 | homogentisate 1,2-dioxygenase | 3.86E-02 | 1.54 |
| Afu6g08210 | GINS DNA replication complex subunit Sld5, putative | 3.43E-02 | 1.54 |
| Afu2g10750 | RNA helicase (Dbp), putative | 4.41E-02 | 1.54 |
| Afu3g03090 | transposase | 2.66E-03 | 1.53 |
| Afu6g08720 | 5'-methylthioadenosine phosphorylase | 3.56E-02 | 1.52 |
| Afu6g07520 | conserved hypothetical protein | 5.59E-03 | 1.52 |
| Afu3g13820 | HET-C2 protein | 1.43E-02 | 1.52 |
| Afu3g14490 | Ketol-acid reductoisomerase | 1.42E-03 | 1.52 |
| Afu5g02150 | proteasome component Pre6, putative | 2.26E-03 | 1.51 |
| Afu5g06430 | mitochondrial large ribosomal subunit L7, putative | 4.95E-02 | 1.51 |
| Afu6g06740 | endoplasmic reticulum calcium ATPase, putative | 3.56E-02 | 1.50 |
| Afu4g07730 | cytosolic large ribosomal subunit L11, putative | 4.45E-04 | 1.50 |
| Afu3g00880 | conserved hypothetical protein | 9.68E-04 | -1.50 |
| Afu2g03300 | hypothetical protein | 1.64E-02 | -1.51 |
| Afu8g01710 | antigenic thaumatin domain protein, putative | 3.67E-03 | -1.54 |
| Afu4g09360 | ATP synthase proteolipid P2, putative | 4.46E-04 | -1.58 |
| Afu4g10440 | conserved hypothetical protein | 1.56E-02 | -1.66 |
| Afu2g11650 | hypothetical protein | 4.41E-02 | -1.66 |
| Afu7g05290 | cytosolic small ribosomal subunit S15, putative | 6.15E-03 | -1.66 |
| Afu8g06450 | iron-sulfur cluster-binding protein, rieske family domain protein | 2.59E-02 | -1.67 |
| Afu6g03790 | hypothetical protein | 1.11E-02 | -1.71 |
| Afu3g07730 | hypothetical protein | 1.87E-02 | -1.74 |
| Afu4g09780 | hypothetical protein | 4.25E-02 | -1.78 |
| Afu5g01000 | oxidoreductase, 2OG-Fe(II) oxygenase family, putative | 3.44E-02 | -1.83 |
| Afu8g07040 | monooxygenase | 2.43E-02 | -1.85 |
| Afu8g05900 | hypothetical protein | 4.78E-02 | -1.95 |
| Afu4g13410 | conserved hypothetical protein | 9.84E-04 | -1.98 |
| Afu7g07170 | reverse transcriptase/protease/endonuclease Pol, putative | 8.03E-03 | -1.98 |
| Afu7g04050 | hypothetical protein | 3.90E-02 | -2.01 |
| Afu3g13780 | hypothetical protein | 2.57E-02 | -2.02 |
| Afu4g08200 | GPI transamidase component PIG-U, putative | 6.62E-03 | -2.10 |
| Afu2g09220 | hypothetical protein | 6.56E-03 | -2.10 |
| Afu6g02310 | hypothetical protein | 2.13E-02 | -2.13 |
| Afu2g16680 | hypothetical protein | 4.80E-02 | -2.16 |
| Afu2g02940 | hypothetical protein | 1.35E-02 | -2.17 |
| Afu5g12710 | SET domain protein | 2.61E-02 | -2.19 |
| Afu4g09420 | Carbonic anhydrase | 2.91E-03 | -2.26 |
| Afu5g10390 | hypothetical protein | 1.09E-02 | -2.32 |
| Afu5g12970 | hypothetical protein | 3.92E-02 | -2.34 |
| Afu4g13640 | hypothetical protein | 2.53E-02 | -2.49 |
| Afu4g08580 | antioxidant protein LsfA | 1.89E-02 | -2.58 |
| Afu4g09920 | conserved hypothetical protein | 1.12E-02 | -2.60 |
| Afu2g11880 | hypothetical protein | 7.29E-04 | -2.63 |
| Afu2g15140 | MSF drug transporter, putative | 1.16E-04 | -3.09 |
| Afu4g04120 | protein kinase activator (Bem1), putative | 5.66E-03 | -3.20 |
